# Effect of Alkali Treatment on Structure and Properties of High Amylose Corn Starch Film

**DOI:** 10.3390/ma12101705

**Published:** 2019-05-26

**Authors:** Yang Qin, Hui Zhang, Yangyong Dai, Hanxue Hou, Haizhou Dong

**Affiliations:** 1 Department of Food Science and Engineering, Shandong Agricultural University, 61 Daizong Street, Tai’an 271000, China; qyghostz@sdau.edu.cn (Y.Q.); zhanghui@sdau.edu.cn (H.Z.); dyyww@sdau.edu.cn (Y.D.); 2 Engineering and Technology Center for Grain Processing of Shandong Province, Tai’an 271000, China

**Keywords:** high amylose starch, alkali treatment, melting extrusion, degradable starch film

## Abstract

Alkali treatment is used for melt extrusion film formation with corn starch, but optimal conditions for this procedure are still unknown. In this study, the changes in properties and structure of high amylose corn starch (70%) films with different concentrations of sodium hydroxide (NaOH), prepared by melting extrusion, were investigated. With increasing sodium hydroxide concentrations, the tensile strength of the high-amylose starch film decreased gradually, while the elongation at break increased. The tensile strength of the high amylose starch (HAS) film with 2% NaOH-treatment was 10.03 MPa and its elongation at break was 40%. A 2% NaOH-treatment promoted the orderly rearrangement of starch molecules and formed an E_h_-type crystal structure, which enlarged the spacing of the single helix structure, increased the molecular mobility of the starch, and slowed down the process of recrystallization; a 10% NaOH-treatment oxidized the hydroxyl groups of the high amylose corn starch during extrusion, formed a poly-carbonyl structure, and initiated the degradation and cross-linking of starch molecule chains.

## 1. Introduction

Petroleum-based plastics have caused serious environmental pollution [1], and thus research into alternatives is needed. Starch, one such alternative, is renewable, biodegradable, non-toxic [2], and is suited for manufacturing thermoplastic film [3]. However, this type of production is not yet proven to be an ecologically sound stock for bioplastics, and their utility requires further research. High amylose corn starch (HAS) possesses a high straight-chain content, short linear-molecular chain length, less branching, tight molecular chain arrangement, and strong intermolecular interaction. Thus, films prepared by HAS show higher mechanical and stronger barrier properties compared to films formed by other starches [4]. However, the dense molecular arrangement and linear molecular structure of HAS also means it has higher thermal stability [5], higher gelatinization temperature, higher melting viscosity, in addition to a faster aging and recrystallization rate [6,7]. These properties make gelatinizing high amylose corn starch difficult, and limit its utility in the preparation of starch films. Although plasticizers can remarkably improve the flexibility and lower gelatinization temperature of starches, they fail to address the disadvantages of HAS films such as brittleness and susceptibility to aging and recrystallization [8].

Alkali treatment is widely used in the process of many traditional foods. A certain level of edible alkali can enhance flour quality and improve the taste and appearance of the product [9,10]. Alkali, especially sodium hydroxide (NaOH), can also aid in the extraction of starch [11]. By soaking raw materials in alkali solution, it can be easy to separate starch from the binding protein, simplify the starch extraction process, and improve the purity of starch products [12,13]. Another important usage of alkali treatment is catalyzing the chemical modification reaction of starch [14]. As most of the chemical modification reactions for starch are nucleophilic reactions, a certain level of alkali treatment can promote a reaction between starch and the modifier by ionizing the hydroxyl groups in the starch molecules into an alkoxide anion (St-O^−^Na^+^). Therefore, alkali is an efficient catalyst for the chemical modification of starch [15,16]. Previous studies have shown that alkali treatment inflates the crystalline region of starch during the extraction and modification process, which benefits starch gelatinization, amylose leaching from the granule [10,17], reduces the proportion of amylopectin in starch [9], and inhibits the occurrence of starch aging recrystallization [18]. Therefore, alkali treatment is a simple and effective method of regulating the crystal structure and processing properties of starch and can be applied to the development of starchy food under various digestibility [19,20] and starch carriers [21].

Current research on alkali-treated starches focuses on the effect of alkali treatment on the structure and properties of starch and the reaction efficiency during the extraction or modification processes. However, the effect of alkali treatment on the properties of thermoplastic starch films prepared by melting extrusion has rarely been reported. In this paper, the properties and structural changes of thermoplastic high amylose corn starch films prepared by melting extrusion after applying different concentrations of sodium hydroxide were studied. The results of our study provide a theoretical basis for the subsequent study of starch extrusion modification and film formation with HAS.

## 2. Materials and Methods

### 2.1. Materials

High amylose corn starch (short as HAS, 70% amylose) was purchased from Ingredion China Limited (Shanghai, China). Dimethyl sulfoxide (DMSO), citric acid, and glycerol (AR) were purchased from Tianjin Kaitong Chemical Reagent Co., Ltd. (Tianjin, China). Deionized water was prepared using an AK Exceed-D lab pure-water system (Chengdu Tangshi Kangning Technology Co., Ltd., Chengdu, China). 

### 2.2. Preparation of Alkali-treated HAS Film

The HAS was placed in a SHR-50A high-speed mixer (Hongji Co., Ltd., Zhangjiagang, China) at pre-determined proportions (Table 1) and slowly sprayed with different concentrations of NaOH solution at 5 Hz. After mixing for 5 min, the homogeneous material was sealed in a Ziploc bag and left at room temperature for 24 h. The alkali-treated HAS was then placed in a high-speed mixer, mixed with 40% glycerol at 15 Hz for 10 min., returned to a Ziploc bag, and left at room temperature for another 24 h. 

The melting extrusion of the HAS was completed by a SHJ-20B co-rotating twin-screw extruder Nanjing Giant Machinery Co., Ltd., Jiangsu, China) equipped with a single hole T-flat die, the length of the die was 40 mm and the diameter of the hole was 3.5 mm. The twin-screw was 20 mm in diameter with its L/D was 40. The extrusion temperature was set from the feeder to the die end: 85-100-115-135-120-115 °C. The extrusion screw speed was 100 rpm and the feeder rotation speed was 10 rpm. These parameters ensured the throughput was about 1 kg/h. An exhaust vent was equipped near the die to for exhausting steam. The extrudate was cooled to room temperature through an air-cooled conveyor belt which consisted of four fans, and cut into strips of 20 cm. Five strips were placed on a 20 cm × 20 cm stainless steel mold covered with tetrafluoroethylene to form a film by hot pressing. The process parameters of the hot pressing film formation were: 155 °C, 0 MPa preheated for 2 min, 10 MPa hot pressed for 10 min, 30 MPa hot pressed for 10 min, circulated water was used to cool the mold to room temperature and open the mold, and the HAS film was placed in an HWS constant-temperature and humidity chamber (Shanghai Jinghong Laboratory Equipment Co., Ltd., Shanghai, China) at 23 °C and 53% RH for 72 h.

### 2.3. Thickness

The thicknesses of the HAS films were determined using 211-101F micrometers (Guilin Guanglu Measuring Instrument Co., Ltd., Guangxi, China). All the films were tested randomly, and the thicknesses of the HAS films were calculated by averaging the results of six replicate measurements. 

### 2.4. Mechanical Properties

The mechanical properties of the starch film including tensile strength (TS, MPa) and elongation at break (EAB, %) were measured by a XLW (PC) Auto Tensile Tester (Labthink Instruments Co., Ltd., Jinan, China) based on the standard of ASTM D882-12 [22]. The test method was as follows: the starch film, preserved at constant temperature and humidity, was cut into strips with 15 cm long and 1.5 cm wide. The test parameters were: a test environment temperature of 23 °C and a relative humidity of 53%. The sample was cut into rectangle strip with 1.5 cm in width and 15 cm in length. A jagged surface clamps were used to prevent the film slipped during the test. The initial distance between the top and bottom clamps was 100 mm and the test speed were 100 mm/min. Each test trial per film consisted of five replicates.

### 2.5. Attenuated Total Reflectance Fourier-Transform Infrared Analysis

Attenuated total reflectance Fourier transform infrared spectroscopy (ATR-FTIR) was performed on the HAS films using a Nicolet iS 5 spectrometers with an attenuated total reflectance accessory (Thermo Fisher Scientific, Waltham, MA, USA) over a wavenumber range of 4000–400 cm^−1^. The number of accumulated scans, the scanning resolution and accumulated scans time were 32, 4 cm^−1^, and 47 s, respectively. The resulting spectra were normalized and difference processed using OMNIC software (Thermo Fisher Scientific, Waltham, MA, USA), and Peakfit software version 4.0 (Systat Software Inc, San Jose, CA, USA) was used to fit the spectra, identify characteristic peaks, and calculate their intensity.

### 2.6. ^13^C Solid-state Nuclear Magnetic Resonance

The ^13^C solid-state nuclear magnetic resonance (^13^C SSNMR) was performed using a Bruker Avance III 400 MHz wide bore spectrometer (Bruker-AXS, Karlsruhe, Germany) equipped with a 4-mm broadband double-resonance cross-polarized magic angle spinning probe. Each film sample (500 mg) was placed onto the rotor and introduced to the center of a magnetic field. The results were normalized and fitted by Peakfit software to calculate the peak areas of C_1_ and C_4_.

### 2.7. Scanning Electron Microscopy

The surface morphology of HAS films was sprayed with gold and measured by a FEI QUANTA FEG25 electron microscope (FEI company, Hillsboro, OR, USA) with a magnification of 2000 times, at an accelerating voltage of 5 KV.

### 2.8. Static and Dynamic Laser Light Scattering 

One mg of the HAS film was dissolved in 1 mL of 50 mM LiCl-DMSO and stirred at 90 °C for 2 h in a water bath. The resulting solution was then magnetically stirred at room temperature for 8 h and injected into a sample vial through a 1.0 μm nylon-66 filter (Jinteng experimental equipment co. Ltd., Tianjin, China).

The average molecular weight of the starch film was measured using a BI-200SM laser light scattering system (Brookhaven Instruments Corporation, NY, USA). The static light scattering (SLS) test had an observation angle of 30–120°, a step frequency of 5°, and the incident laser wavelength was λ = 633 nm. The refractive index of the sample solution was measured using a dn/dc meter to be 0.147. The test results were calculated by the Berry equation to obtain the average molecular weight (M_w_) and radius of gyration (R_g_) of the HAS film.

The dynamic light scattering (DLS) test had an observation angle of 30–120°, a step frequency of 10°, test time of 3 min (for a total duration of 30 min) and an incident laser wavelength of λ = 633 nm. The experimental results were calculated by the Contin equation, and the effective particle size of the starch molecules at various angles was identified. The hydrodynamic radius (R_h_) was obtained by mathematical derivation, and by calculating the R_g_/R_h_ ratio, the morphology of the starch molecules in the solution was identified.

### 2.9. X-ray Diffraction

The crystallinity and crystal structure of the HAS film were tested by X-ray diffraction (XRD) using a D8 Advance X-ray diffractometer (Bruker-AXS, Karlsruhe, Germany). The Cu target line was used, λ = 0.15406 nm, 2θ = 5–40°, and the step frequency was 0.02°/s.

### 2.10. Statistical Analysis

Data are expressed as mean ± standard deviation, and differences were considered significant at *p* < 0.05, after analysis of variance (ANOVA) with SPSS software 21 (IBM Co., NY, USA).

## 3. Results and Discussion

### 3.1. Effect of Alkali Treatment on the Appearance of HAS film

**Figure 1:**
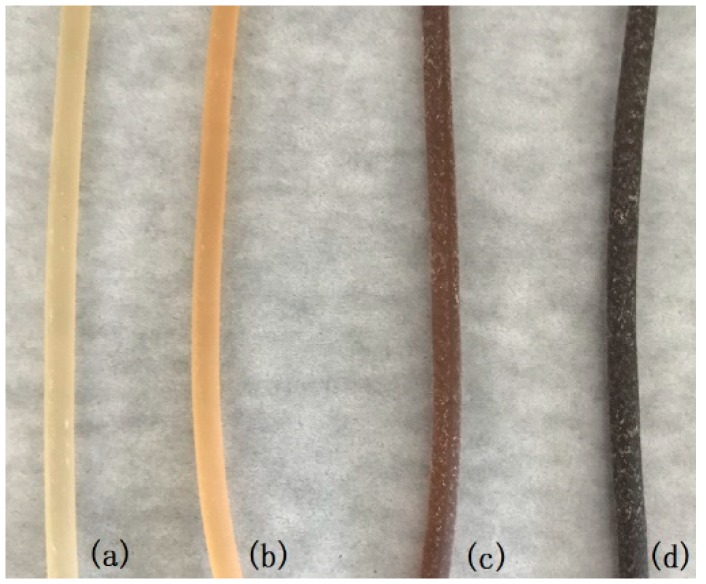
Appearance of HAS extrudates treated with different concentrations of NaOH. (**a**) Control; (**b**) 2% NaOH; (**c**) 6% NaOH; and (**d**) 10% NaOH.

As the concentration of NaOH increased, the melting extrusion process caused the color of the thermoplastic HAS to gradually darken from a translucent yellow to a dark brown (Figure 1). The surface of the control group was smooth with a small number of white spots; the surface of HAS treated with 10% NaOH concentration was rough, and irregular aggregation of starches was observed. This may be caused by the oxidation of starch molecules, molecular chain breaks, and random cross-linking after the NaOH treatment.

### 3.2. Effect of Alkali Treatment on Mechanical Properties of HAS Film

**Figure 2:**
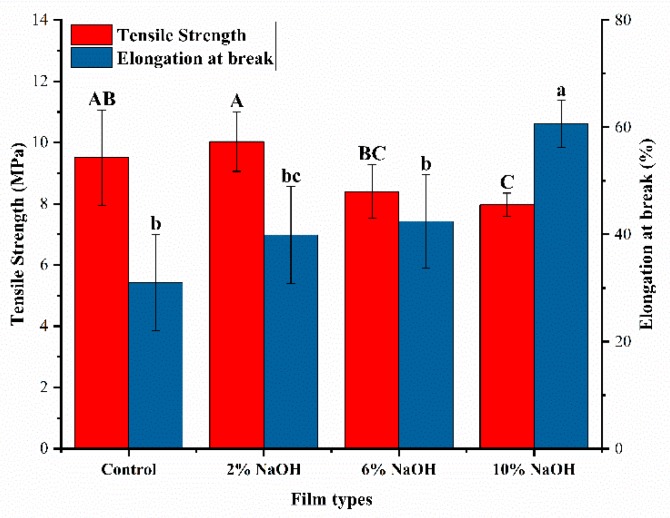
Tensile strength and elongation at break of HAS films treated with different concentrations of NaOH. Different letters in the same column represent significant differences, *p* < 0.05.

NaOH treatment increased the elongation at break, but decreased the tensile strength of HAS film as the NaOH concentration increased (Figure 2). The tensile strength of the control group was 9.51 MPa and the elongation at break was 31%. The tensile strength of the HAS film with 2% NaOH slightly increased to 10.03 MPa and its elongation at break increased to 40%. The increased tensile strength and elongation at break can be attributed to low concentration alkali promoting gelatinization of HAS, which facilitated the leaching of amylose from the crystalline structure, formed more hydrogen bonds with the amorphous region, and enhanced its tensile strength. NaOH also rearranged the molecular chain of HAS. This changed the crystal structure of starches, increased the space within starch molecular chains, and improved the elongation at break [23]. The 10% NaOH-treated HAS film had the lowest tensile strength of 7.96 MPa and the highest elongation at break of 60%. This could be due to the fact that, during the melting extrusion process, high concentrations of NaOH oxidized the anhydroglucose of some starches and caused irregular cross-linking of the starch molecular chains, increasing the branching degree and molecular gap of the starch chain, resulting in increased flexibility [3]. This reduced the tensile strength of the HAS film and enhanced its toughness [24].

### 3.3. Effect of Alkali Treatment on Thickness of HAS Film

**Figure 3:**
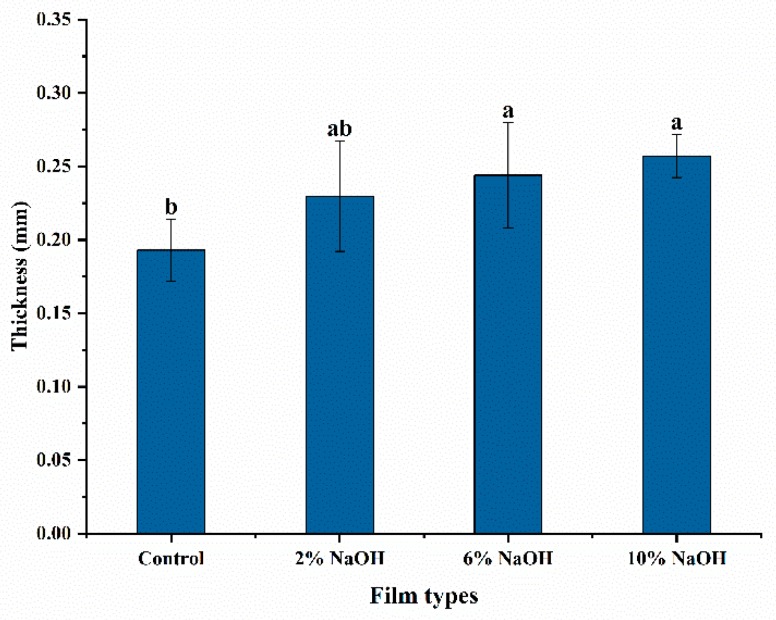
Thickness of HAS film after treatment with different concentrations of NaOH. Different letters in the same column represent significant differences, *p* < 0.05.

The thickness of the control group was 0.193 mm. The thickness of HAS treated with 2%, 6%, and 10% NaOH was 0.229 mm, 0.249 mm, and 0.257 mm, respectively (Figure 3).

### 3.4. Effect of Alkali Treatment on the Morphology of HAS Film

**Figure 4:**
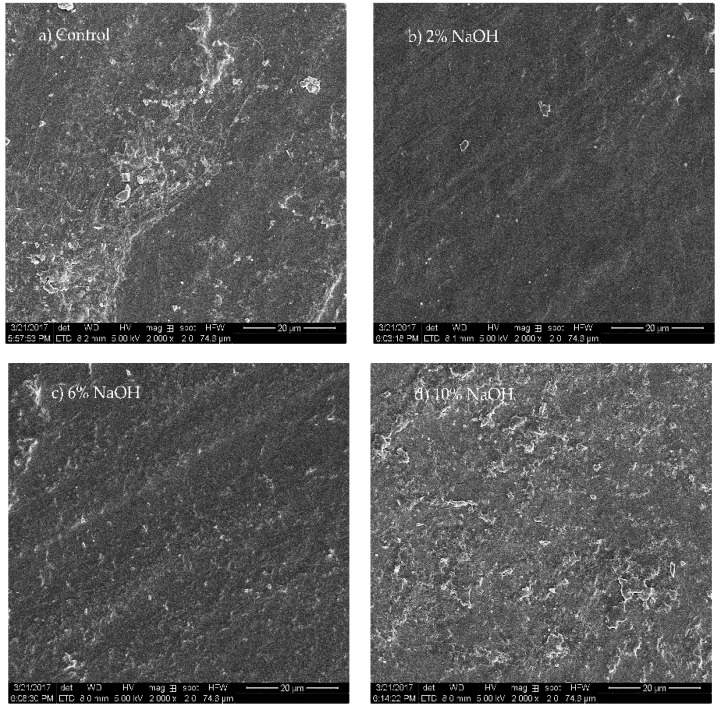
Surface morphology of HAS films treated with different concentrations of NaOH. (**a**) control; (**b**) 2% NaOH; (**c**) 6% NaOH; (**d**) 10% NaOH.

The HAS films treated with different NaOH concentrations showed significantly different surface morphologies (Figure 4). The surface of the control group was rough with a large number of particles. The main source was the HAS residue that was not fully gelatinized during extrusion and subsequent recrystallization after cooling [25]. The surface of the HAS film treated with 2% NaOH was smooth and flat, and the number of particles was significantly reduced. This indicates that 2% NaOH promoted gelatinization of the HAS in the film formation, effectively reducing the retention of starch residues, and inhibiting the recrystallization of HAS. With increasing NaOH concentrations, the surface of HAS films with 6% NaOH and 10% NaOH became rough with irregular starch aggregations. Higher NaOH concentrations led to oxidative degradation of the starch molecules, and the formation of a large number of small molecular fragments and irregularly cross-linked starch chains, none of which were conducive to the formation of a uniform surface [10].

### 3.5. Effect of Alkali Treatment on the Crystal Structure of HAS Film

**Figure 5:**
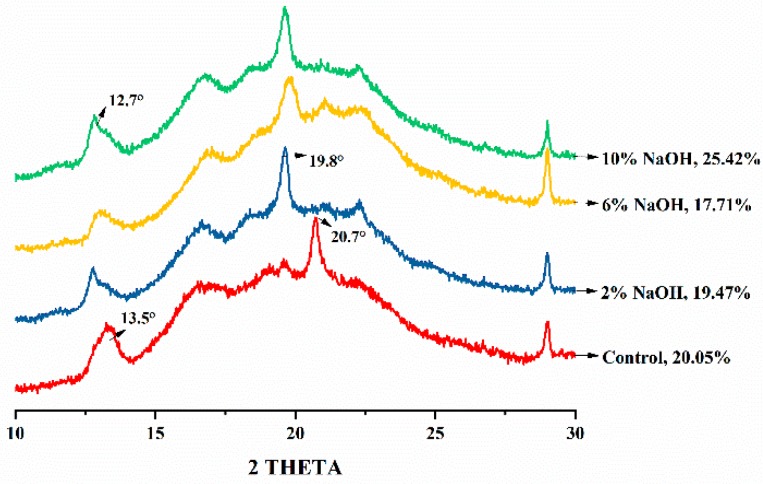
XRD patterns of HAS films treated with different concentrations of NaOH. The percentage in the figure is the crystallinity of the films.

The X-ray diffraction (XRD) pattern of the control group showed a distinct V_H_-type crystal structure (Figure 5), which had a strong diffraction peak at 2θ = 13.5° and 20.7°, and a weak diffraction peak at 2θ = 19° [26]. This typical V_H_-type crystallization indicates that after extrusion and hot-press into a film, HAS starch formed a regularly ordered crystal structure mainly composed of amylose in a single helix structure [27,28]. Although the crystal structure of the HAS film with 2% NaOH also belongs to the V-type crystal structure, the crystal peak at 2θ = 13.5° lowered the angle to 12.7°, the strong peak at 2θ = 20° changed to a weak peak, and a strong diffraction peak appeared at 2θ = 19°. NaOH changed the crystalline conformation of amylose molecules, which promoted the conversion of V_H_-type starch crystals into E_H_-type crystals [10,29]. This unstable crystal structure of the amylose has the characteristics of a small single helix pitch, large space inside the helix structure as well as an increased molecular chain gap [28], which improved the relative movement of the starch molecular chain and increased the elongation at break of the HAS film. Furthermore, it can form more hydrogen bonds with water molecules, maintaining the tensile strength of the HAS film [23]. The characteristic peak of the 6% NaOH-treated HAS film showed a decrease in intensity and an increase in the half-peak width. This indicates that the increased concentration of NaOH contributed to the gelatinization of starch, that the E_H_-type crystal structure of the starch was destroyed by NaOH, and the resulting amylose leaching and formation of an amorphous structure [30] led to a decrease in tensile strength of the HAS film. However, the crystallinity of the HAS film treated with 10% NaOH was 25.42%, which increased compared to the control that was 20.05%. This could be attributed to the fact that a high concentration of NaOH induces oxidation and decomposition of the amorphous zone in the starch, resulting in an increase in the proportion of the crystallization zone during the extrusion process [31].

### 3.6. Effect of Alkali Treatment on the Molecular Structure of HAS Film

**Figure 6:**
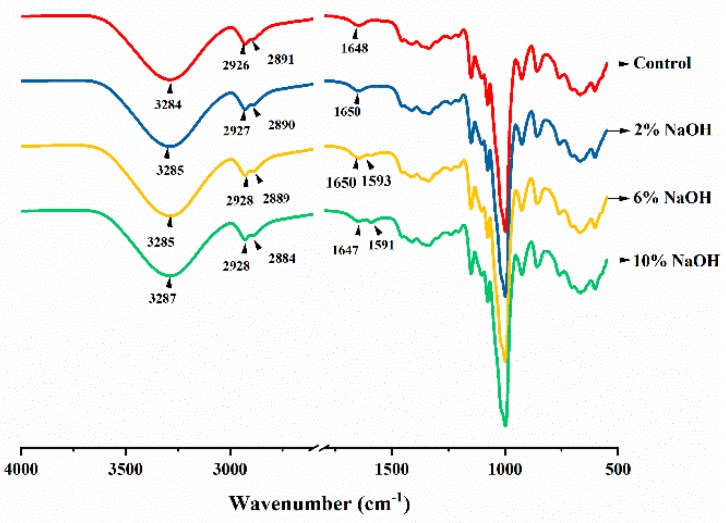
FTIR spectra of HAS films treated with different concentrations of NaOH.

The Fourier transform infrared (FTIR) spectrum of the HAS film with different concentrations of NaOH (Figure 6) yielded the following results: the broad peak near 3280 cm^−1^ was the stretching vibration peak of -OH in the starch film, while the bending vibration peak of -OH at 1650 cm^−1^ was the characteristic absorption peak of water molecules. The double peaks located near 2925 cm^−1^ and 2881 cm^−1^ were symmetric and asymmetric stretching vibration peaks of -CH_2_ and -CH, and their bending vibration peaks were located at 1365 cm^−1^, 1235 cm^−1^, and 1200 cm^−1^. The vibrational peak of the glucose ring was located at 925 cm^−1^, and the stretching vibrational peak of the C-O-C group of the α-1,4-glycosidic bond was located near 1000 cm^−1^ [32].

Compared to the control group, the absorption peak intensity of the 2% NaOH-treated HAS film at 3285 cm^−1^ increased. This was mainly due to the reaction of NaOH with the -OH group, which formed an -O^−^Na^+^ group, destroying the hydrogen bond structure that maintains the structural stability of the starch molecule and exposing more -OH groups [33]. When the concentration of added NaOH increased to 6% and 10%, the intensity of the absorption peak of -OH near 3285 cm^−1^ decreased due to the reduced content of -OH after oxidization. The characteristic peaks at 2900–2850 cm^−1^ and 1500–1183 cm^−1^ showed similar trends. The presence of -O^−^Na^+^ enhanced the polarity of -CH and -CH_2_, resulting in enhanced absorption peaks at 2% NaOH-treatment; as the NaOH concentration further increased, the starch molecules oxidize, resulting in a decrease in the absorption peaks of -CH and -CH_2_.

The oxidization of HAS during extrusion occurred in the HAS film with 6% NaOH, forming a new vibrational absorption peak of carbonyl (C=O) around 1591 cm^−1^, indicating that the -OH group in anhydroglucose was oxidized [34]. In the HAS film with 10% NaOH the intensity of its associated characteristic peak increased, indicating that NaOH promotes the oxidation reaction of starch.

**Figure 7:**
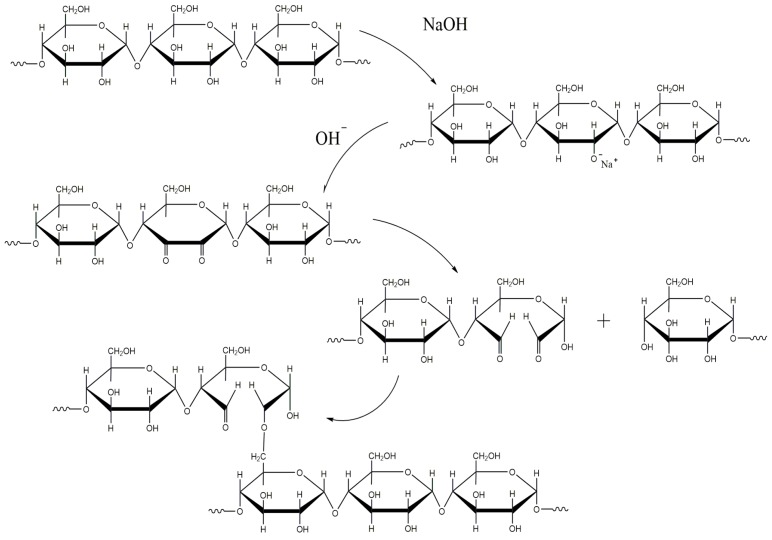
Hypothesis of chemical reaction of alkali treated HAS during extrusion.

The current explanation of the oxidation process of alkali-treated HAS in the extrusion process was based on the study of the degradation mechanism of cellulose by alkali solution [35] (Figure 7). NaOH caused the HAS to expose more hydroxyl reduction ends by swelling starch granules and forming -O^−^Na^+^ groups. Under high temperatures and high shear stress conditions, NaOH reacted with the hydroxyl group of the starch molecule and formed a molecule containing a di-carbonyl group via molecular rearrangement [33]. In addition to oxidizing the hydroxyl groups of the starch, NaOH also weakened the absorption peak of the C-O-C bond of the starch molecule near 1000 cm^−1^, which increased the band intensity at 800–500 cm^−1^. This indicates that during the melting extrusion process, NaOH initiated the degradation of starch molecules, broke the glycosidic bond, and produced a small molecule of carboxylic acid molecules or heterocyclic rings containing an unsaturated bond [35]. The HAS film molecular weights indicate that 10% NaOH-treated HAS also underwent a cross-linking reaction during the extrusion film formation process.

Analysis of the short-range order of molecular chains in HAS films after treatment with varying concentrations of NaOH by FTIR further explored the molecular chain changes of starch during film formation [36]. When HAS is extruded and hot pressed into a film, the hydrogen bonding between adjacent molecules changed due to thermal effects and shear stress, causing the relative characteristic peaks in the 1200–900 cm^−1^ band to move to lower wavenumbers [37]. Therefore, for the NaOH-treated HAS film prepared by melt extrusion, the evaluation index of the short-range order of the molecular chain was evaluated as the peak intensity ratio at 1039 cm^−1^ and 1018 cm^−1^ (R_1039/1018_).

The R_1039/1018_ values of the HAS films treated with different concentrations of NaOH showed a tendency to first increase and then decrease, with a maximum value of about 0.707 at 6% NaOH (Table 2). This indicates that when the concentration of NaOH was less than 6%, NaOH affected HAS mainly by promoting the precipitation of amylose and the rearrangement of molecular chains. When the amount of NaOH reached 10%, the R_1039_/_1018_ value was only 0.465, indicating that the rearrangement of the NaOH starch molecular chain occupied a secondary position and destroyed the short-range order of starch molecules by alkaline hydrolysis, oxidation, and cross-linking, which caused a loose and irregular structure of starch chains.

### 3.7. Effect of Alkali Treatment on Molecular Chain Conformation of HAS Film

**Figure 8:**
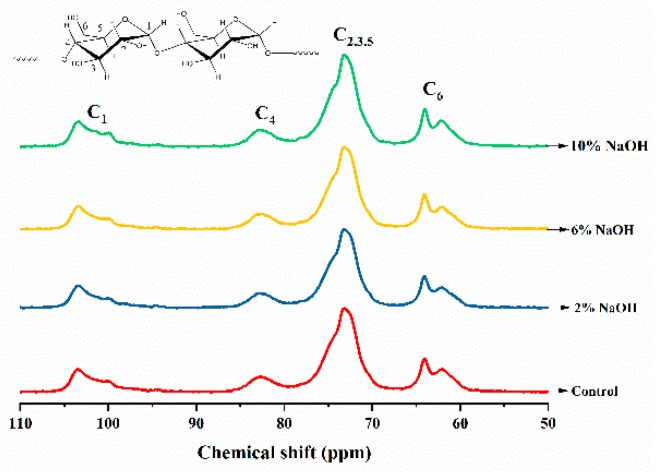
^13^C solid-state NMR spectra of HAS films treated with different concentrations of NaOH.

The ^13^C solid-state nuclear magnetic resonance (NMR) spectra of HAS films treated with different concentrations of NaOH (Figure 8) showed that the characteristic peak of C_1_ was located at 90–110 ppm, in which 99–101 ppm peak represented the double helix structure of HAS molecular chain and 102–103 ppm peak represented the single helix moiety in the starch granules. The characteristic peak of C_4_ was located at 78–88 ppm and its peak area ratio was related to the ratio of the amorphous zone in HAS film; the strong peak at 68–78 ppm was the characteristic peak produced by the interaction of C_2_, C_3_, and C_5_; and the characteristic peak of C_6_ was located at 58–68 ppm [38].

The change in molecular chain conformation of the HAS films treated with different concentrations of NaOH was measured by fitting the ^13^C solid-state NMR spectra (Table 3). Compared with the control group, the ratio of the double helix structure in the HAS film treated with 2% and 6% NaOH was significantly reduced and the proportion of the amorphous region increased, indicating that NaOH effectively promoted gelatinization of HAS during extrusion and inhibited the occurrence of recrystallization after film formation. In the HAS film with 10% NaOH, the proportion of double helix structures increased, and the proportion of single helix structures and amorphous regions decreased. Together with the XRD and FTIR results, this indicates that in the 10% NaOH-treated HAS film, a partial double helical array of starch chains similar to crystalline structures with a loose structure was present [39], which may be the result of recrystallization of starch fragments or cross-linked starch chains.

### 3.8. Effect of Alkali Treatment on the Average Molecular Weight of HAS Film

The molecular weight and molecular morphology of HAS films with different concentrations of NaOH changed significantly (Table 3). The molecular weight of the control group was 2.69 ± 0.29 × 10^5^ g·mol^−1^, and its R_g_/R_h_ value was 0.97, indicating that the starch molecules were hollow sphere structures with a dense shell and a relatively loose interior. This demonstrates that HAS maintained a tight outer shell after extrusion film formation, and the use of water/glycerol as a plasticizer is ineffective on HAS. The R_h_ of the HAS film treated with 2% and 6% NaOH increased gradually, the R_g_ decreased gradually, the R_g_/R_h_ decreased, HAS particles retained a hard sphere state with a dense core remaining, while the outer shell became gradually looser, and the molecular gap increased. This demonstrates that the swelling of HAS particles caused by NaOH progresses from the outside to the inside. When the amount of NaOH reached 10%, the R_g_/R_h_ of the HAS molecule increased to 1.20, indicating that the form of the HAS molecule in solution was a random coil [40] and that NaOH destroyed the structure of the starch granule.

The molecular weight of HAS gradually increased as the NaOH concentration increased, and the molecular weight of 10% NaOH-treated HAS film was 1.84 times that of the control group. Starch molecules may not only undergo an oxidative decomposition reaction, but also undergo a cross-linking reaction during the HAS extrusion process with high concentration NaOH treatment [41]. The substance that initiated starch cross-linking may be a small molecule like a carboxylic acid or an aldehyde group produced by alkaline hydrolysis, or a starch chain containing a dialdehyde group. Irregular cross-linking resulted in random aggregation of starch and an uneven distribution of starch molecular chains. This may be the underlying cause of rough surfaces, reduced tensile strength, increased elongation at break, and increased thickness after film formation.

## 4. Conclusions

The study fills a research gap in the existing literature by demonstrating that treatment with alkali can affect the properties and performance of corn starch films. The data showed that as the dosage of NaOH increased, the tensile strength of HAS film decreased and the elongation at break increased gradually. A 2% NaOH-treatment HAS had a tensile strength at 10.03 MPa and its elongation at break was 40%. The 2% NaOH orderly rearranged the starch molecules, formed E_h_-type crystal structures, increased the spacing in a single-helical structure, increased the mobility of starch molecules, and slowed the process of recrystallization. This would be beneficial for the further modification and film forming process of HAS.

As the concentration increased, especially 10% NaOH, alkali oxidized the hydroxyl group of HAS significantly during extrusion and formed poly-carbonyl groups and small molecules. These might initiate various cross-linking of starch that increased the molecular weight to 4.97 × 10^5^ g·mol^−1^ and caused random aggregation of the starch molecular chain, led to recrystallization of starch, and browned the starch film, which were not conducive to the further modification of starch and starch film performance improvement. These results provide a guiding framework for further investigation into HAS modification by extrusion and film formation.

## Figures and Tables

**Table 1:** Raw material ratios of HAS film with different NaOH additions.

Sample Name	HAS/kg	Glycerin/g	Water/g	NaOH/g
Control	2	800	600	0
2% NaOH	2	800	600	40
6% NaOH	2	800	600	120
10% NaOH	2	800	600	200

**Table 2:** R_1039/1018_ values, single and double helix structures, and amorphous area peak area ratio of HAS films treated with different concentrations of NaOH.

Sample Names	R_1039/1018_	Double Helix Zone /%	Single Helix Zone /%	Amorphous Zone /%
Control	0.502	22.42	37.12	40.46
2% NaOH	0.693	19.37	36.79	43.84
6% NaOH	0.707	19.39	37.40	43.20
10% NaOH	0.465	27.79	31.98	40.24

**Table 3:** Average molecular weight and R_g_/R_h_ value of HAS films treated with different concentrations of NaOH.

Sample Name	R_h_ /nm	R_g_ /nm	R_g_/R_h_	M_w_ /×10^5^ g·mol^−1^
Blank Control	128.43	124.60 ± 7.0	0.97	2.69 ± 0.29
2% NaOH	148.71	119.00 ± 6.2	0.80	2.63 ± 0.25
6% NaOH	152.37	119.50 ± 5.2	0.78	3.07 ± 0.25
10% NaOH	121.33	146.10 ± 6.4	1.20	4.97 ± 0.43

## References

[1] Khanoonkon N., Yoksan R., Ogale A.A. (2016). Morphological characteristics of stearic acid-grafted starch-compatibilized linear low density polyethylene/thermoplastic starch blown film. Europ. Polym. J..

[2] Tena-Salcido C.S., Rodríguez-González F.J., Méndez-Hernández M.L., Contreras-Esquivel J.C. (2008). Effect of morphology on the biodegradation of thermoplastic starch in ldpe/tps blends. Polym. Bull..

[3] Versino F., Lopez O.V., Garcia M.A., Zaritzky N.E. (2016). Starch-based films and food coatings: An overview. Starch Stärke.

[4] Li M., Liu P., Zou W., Yu L., Xie F., Pu H., Liu H., Chen L. (2011). Extrusion processing and characterization of edible starch films with different amylose contents. J. Food Eng..

[5] Ratnayake W.S., Jackson D.S. (2008). Chapter 5 starch gelatinization. Adv. Food Nutr. Res..

[6] Liu P., Xie F., Li M., Liu X., Yu L., Halley P.J., Chen L. (2011). Phase transitions of maize starches with different amylose contents in glycerol–water systems. Carbohydr. Polym..

[7] Wang J., Yu L., Xie F., Chen L., Li X., Liu H. (2010). Rheological properties and phase transition of cornstarches with different amylose/amylopectin ratios under shear stress. Starch Stärke.

[8] Kim H., Jane J., Lamsal B. (2017). Hydroxypropylation improves film properties of high amylose corn starch. Ind. Crops Prod..

[9] Cai J., Yang Y., Man J., Huang J., Wang Z., Zhang C., Gu M., Liu Q., Wei C. (2014). Structural and functional properties of alkali-treated high-amylose rice starch. Food Chem..

[10] Karim A.A., Nadiha M.Z., Chen F.K., Phuah Y.P., Chui Y.M., Fazilah A. (2008). Pasting and retrogradation properties of alkali-treated sago (metroxylon sagu) starch. Food Hydrocoll..

[11] Han X., Hamaker B.R. (2002). Partial leaching of granule-associated proteins from rice starch during alkaline extraction and subsequent gelatinization. Starch Stärke.

[12] Ragheb A.A., Abdel-Thalouth I., Tawfik S. (1995). Gelatinization of starch in aqueous alkaline solutions. Starch Stärke.

[13] Dokić L., Dapčević T., Krstonošić V., Dokić P., Hadnađev M. (2010). Rheological characterization of corn starch isolated by alkali method. Food Hydrocoll..

[14] Gray J.A., BeMiller J.N. (2004). Development and utilization of reflectance confocal laser scanning microscopy to locate reaction sites in modified starch granules. Cereal Chem..

[15] Masina N., Choonara Y.E., Kumar P., du Toit L.C., Govender M., Indermun S., Pillay V. (2017). A review of the chemical modification techniques of starch. Carbohydr. Polym..

[16] Li Y., Lim S. (2016). Preparation of aqueous alpha-lipoic acid dispersions with octenylsuccinylated high amylose starch. Carbohydr. Polym..

[17] Wang S., Copeland L. (2012). Effect of alkali treatment on structure and function of pea starch granules. Food Chem..

[18] Israkarn K., Na Nakornpanom N., Hongsprabhas P. (2014). Physicochemical properties of starches and proteins in alkali-treated mungbean and cassava starch granules. Carbohydr. Polym..

[19] Han J., Lim S. (2004). Structural changes in corn starches during alkaline dissolution by vortexing. Carbohydr. Polym..

[20] Qiao D., Yu L., Liu H., Zou W., Xie F., Simon G., Petinakis E., Shen Z., Chen L. (2016). Insights into the hierarchical structure and digestion rate of alkali-modulated starches with different amylose contents. Carbohydr. Polym..

[21] Tamaddon F., KazemiVarnamkhasti M. (2017). Scalable preparation, characterization, and application of alkali-treated starch as a new organic base catalyst. Carbohydr. Res..

[22] ASTM (2012). D882-12 Standard Test Method for Tensile Properties of Thin Plastic Sheeting.

[23] van Soest J.J.G., Vliegenthart J.F.G. (1997). Crystallinity in starch plastics: Consequences for material properties. Trends Biotechnol..

[24] Koch K., Gillgren T., Stading M., Andersson R. (2010). Mechanical and structural properties of solution-cast high-amylose maize starch films. Int. J. Biol. Macromol..

[25] Thiré R.M.S.M., Simão R.A., Andrade C.T. (2003). High resolution imaging of the microstructure of maize starch films. Carbohydr. Polym..

[26] Wang S., Li C., Copeland L., Niu Q., Wang S. (2015). Starch retrogradation: A comprehensive review. Compr. Rev. Food Sci. Food Saf..

[27] Ricciardi R., D’Errico G., Auriemma F., Ducouret G., Tedeschi A.M., De Rosa C., Lauprêtre F., Lafuma F. (2005). Short time dynamics of solvent molecules and supramolecular organization of poly (vinyl alcohol) hydrogels obtained by freeze/thaw techniques. Macromolecules.

[28] Le Bail P., Bizot H., Ollivon M., Keller G., Bourgaux C., Buléon A. (1999). Monitoring the crystallization of amylose–lipid complexes during maize starch melting by synchrotron x-ray diffraction. Biopolymers.

[29] Shi R., Zhang Z., Liu Q., Han Y., Zhang L., Chen D., Tian W. (2007). Characterization of citric acid/glycerol co-plasticized thermoplastic starch prepared by melt blending. Carbohydr. Polym..

[30] Nor Nadiha M.Z., Fazilah A., Rajeev B., Aliasa K. (2010). Comparative susceptibilities of sago, potato and corn starches to alkali treatment. Food Chem..

[31] Li M., Hasjim J., Xie F., Halley P.J., Gilbert R.G. (2014). Shear degradation of molecular, crystalline, and granular structures of starch during extrusion. Starch Stärke.

[32] Lammers K., Arbuckle-Keil G., Dighton J. (2009). Ft-ir study of the changes in carbohydrate chemistry of three new jersey pine barrens leaf litters during simulated control burning. Soil Biol. Biochem..

[33] Tang M., Wen S., Liu D. (2016). Effects of heating- or caustic-digested starch on its flocculation on hematite. Miner. Process. Extr. Metall. Rev..

[34] Shi A.-M., Li D., Wang L.-J., Li B.-Z., Adhikari B. (2011). Preparation of starch-based nanoparticles through high-pressure homogenization and miniemulsion cross-linking: Influence of various process parameters on particle size and stability. Carbohydr. Polym..

[35] Golova O.P., Nosova N.I. (1973). Degradation of cellulose by alkaline oxidation. Russ. Chem. Rev..

[36] Sevenou O., Hill S.E., Farhat I.A., Mitchell J.R. (2002). Organisation of the external region of the starch granule as determined by infrared spectroscopy. Int. J. Biol. Macromol..

[37] Spiridon I., Teaca C.-A., Bodirlau R. (2011). Preparation and characterization of adipic acid-modified starch microparticles/plasticized starch composite films reinforced by lignin. J. Mater. Sci..

[38] Tan I., Flanagan B.M., Halley P.J., Whittaker A.K., Gidley M.J. (2007). A method for estimating the nature and relative proportions of amorphous, single, and double-helical components in starch granules by ^13^c cp/mas nmr. Biomacromolecules.

[39] Qin Y., Liu C., Jiang S., Xiong L., Sun Q. (2016). Characterization of starch nanoparticles prepared by nanoprecipitation: Influence of amylose content and starch type. Ind. Crops Prod..

[40] Wang X., Wu C. (1999). Light-scattering study of coil-to-globule transition of a poly(n-isopropylacrylamide) chain in deuterated water. Macromolecules.

[41] Menzel C., Olsson E., Plivelic T.S., Andersson R., Johansson C., Kuktaite R., Jarnstrom L., Koch K. (2013). Molecular structure of citric acid cross-linked starch films. Carbohydr. Polym..

